# Conceptual and methodological issues in the study of the personality-and-culture relationship

**DOI:** 10.3389/fpsyg.2023.1077851

**Published:** 2023-03-28

**Authors:** Jüri Allik, Anu Realo, Robert R. McCrae

**Affiliations:** ^1^Institute of Psychology, University of Tartu, Tartu, Estonia; ^2^Department of Psychology, University of Warwick, Coventry, United Kingdom; ^3^Retired, Gloucester, MA, United States

**Keywords:** culture, personality, Five-Factor Theory, characteristic adaptations, basic traits, convergent and discriminant validity

## Abstract

Culture-and-personality studies were central to social science in the early 20th century and have recently been revived (as personality-and-culture studies) by trait and cross-cultural psychologists. In this article we comment on conceptual issues, including the nature of traits and the nature of the personality-and-culture relationship, and we describe methodological challenges in understanding associations between features of culture and aspects of personality. We give an overview of research hypothesizing the shaping of personality traits by culture, reviewing studies of indigenous traits, acculturation and sojourner effects, birth cohorts, social role changes, and ideological interventions. We also consider the possibility that aggregate traits affect culture, through psychological means and gene flow. In all these cases we highlight alternative explanations and the need for designs and analyses that strengthen the interpretation of observations. We offer a set of testable hypotheses based on the premises that personality is adequately described by Five-Factor Theory, and that observed differences in aggregate personality traits across cultures are veridical. It is clear that culture has dramatic effects on the thoughts, feelings, and behaviors from which we infer traits, but it is not yet clear whether, how, and in what degree culture shapes traits themselves.

## Conceptual and methodological issues in the study of the personality-culture-relationship

After a period of neglect in the second half of the 20th century, the relationship between personality and culture has again become a popular topic (McCrae and Allik, 2002; Triandis and Suh, 2002; Diener et al., 2003; Benet-Martínez and Oishi, 2008; Heine and Buchtel, 2009; Church, 2017; Allik and Realo, 2019; Draguns and Tanaka-Matsumi, 2020; Lu et al., 2023). Although other variants have been discussed, many researchers were inclined to accept the premise that personality is made from a malleable substance which can be shaped and molded by external forces, including those that spring from culture (e.g., Bleidorn et al., 2019). However, there is a possibility that not all parts of personality are equally susceptible to cultural and other external influences.

This article is not an attempt to compile a state-of-the-art review of what we know about how culture can or cannot influence personality. We are more interested in the logical and methodological issues that are raised by culture-and-personality relationships. What are the issues in the definition of personality? In the assessment of traits? In cross-cultural comparisons? In causal analysis? Many of these methodological questions have been ignored, with the result that many deceptively easy answers have been uncritically accepted. Thus, instead of an exhaustive review of literature our goal is to propose more rigorous guidelines for the study of culture-and-personality relationships.

Historically, personality-and-culture studies were guided chiefly by psychodynamic theories (Malinowski, 1927; Whiting and Child, 1953), and any theory of personality—self-determination (Deci and Ryan, 2000), social-cognitive (Cervone, 2005), or evolutionary (Buss, 1991), just to name a few—could be examined in relation to culture. Even so, in most of what follows we are concerned with a trait model of personality, which is the dominant paradigm in personality psychology and widely used in studies of personality-and-culture.

## Conceptual models

To begin with, the principal schemes describing the relationship between culture and personality need to be distinguished.

### Culture and personality belong to the same category

There is a view that culture and personality belong to the same semantic, that the products of the human mind and culture are inseparable and mutually constitutive (e.g., Shweder, 1991, 1999). One version of this inseparability concept maintains that as soon as language and other cultural sign systems contact something—including personality—this automatically becomes a part of culture (e.g., Luria, 1976). However, if we accept a theoretical position according to which there are no separate “culture things” and “personality things” we will be not able to study how they are related (McCrae, 2009; Allik and Realo, 2019). We cannot even talk about them as two different entities (cf. Shweder, 1999) or ask whether one depends on or influences the other (cf. McCrae, 2009). This position is axiomatic and not subject to empirical test.

In contrast, most researchers in this field assume that culture and personality are separable entities. For this article, we will assume that there is a general consensus on what culture is, but will call attention to different views of personality that have important implications for interpreting the relationship. We have already noted that we will focus on personality as a system of traits, but we will argue that there are at least two different views of traits. In one, traits are construed as the set of habits, styles, interests, and so on that characterize the individual over relatively long periods of time; we call this the *phenotypic theory of traits*. In the other, traits are seen as underlying dispositions that shape phenotypic traits. This genotypic theory is represented by Five-Factor Theory (FFT; McCrae and Costa, 1999, 2008).

### Culture determines personality

As already mentioned, a popular view among personality psychologists is that culture can shape, program, or at least modify human personality (cf. Triandis and Suh, 2002; Benet-Martínez and Oishi, 2008; Heine and Buchtel, 2009; Smaldino et al., 2019; Lu et al., 2023). This is obviously a continuation of what was dubbed the *Standard Social Science Model* (Barkow et al., 1992) with the main emphasis on cultural determinism. One famous example of this approach is Ruth Benedict’s *Patterns of Culture*, which maintains that Dobuans of Melanesia were raised to become suspicious and paranoid, the Kwakiutls were nurtured as autocrats and despots, and the Pueblos of New Mexico were raised to become unemotional and passive as a consequence of their parenting practices (Benedict, 1934/1959).

One of the most influential papers on the personality-and-culture relationship was published by Markus and Kitayama (1991), who showed that people in different cultures have vastly different self-construals, as for example when they answer the question “Who am I?” 20 times. With an independent self, “interaction with others (actual, imagined, or implied) produces a sense of self as separate, distinct, or independent from others” while with interdependent self, “interaction with others produces a sense of self as connected to, related to, or interdependent with others. These interactions are guided by culturally prescribed tasks that require and encourage fitting in with others” (Markus and Kitayama, 2010, p. 423). In other words, culture influences how people view themselves, and how people view themselves may determine their behavior. Thus, culture provides scripts and scenarios, which people learn to think and speak about themselves.

Note that this model does not distinguish between phenotypic and genotypic traits.

### Culture influences only the expression of personality

It has been proposed in FFT that cultural variation influences one part of personality without affecting another part. The distinction between two parts of personality, a tangible and a more abstract one, was not new. Allport (1966) had proposed that we can see cognitive, emotional, or behavioral indicators of various personality dispositions, but not traits themselves or their constitutive mechanisms (p. 3). In FFT these two aspects of personality are called *characteristic adaptations* (the phenotypic indicators of traits) and *basic tendencies* (the genotypic dispositions), and traits are viewed as basic tendencies. What was novel in FFT was the claim that traits were not merely unobservable, they were also essentially independent of external influences; only characteristic adaptations could be shaped by the environment.

This premise was initially based on demonstrations of the remarkable stability of personality traits (McCrae et al., 1980) despite the accumulation of life experiences. It was also supported by other lines of evidence, such as the repeated finding of behavioral genetic studies that indicated substantial heritability for all traits, and a lack of influence from the environment shared by children in the same family. The isolation of traits from environmental influences had profound consequences for personality-and-culture: It implied that basic trait psychology would be much the same everywhere. That prediction was supported by the ease of developing valid translations of personality questionnaires and by cross-cultural studies showing the near-universality of trait structure, age differences, gender differences, and psychometric properties (McCrae and Costa, 1997; Allik et al., 2013). Church et al. (2008) showed that traits functioned similarly in collectivistic and individualistic cultures.

FFT acknowledges the obvious fact that people’s interests, habits, attitudes, and so on vary across time and cultures, but it claims that these features of personality are not traits—they are acquired characteristic adaptations. Characteristic adaptations are shaped in part by traits, and in consequence, they can serve as trait indicators. Having a wide circle of friends is not a trait, but it is a sign of extraversion. Culture can shape one’s friendship network (e.g., women are not allowed to have opposite-sex friends in some traditional cultures), but this is quite different from shaping extraversion itself.

Although the components of FFT are common to many personality theories, the claim that basic traits are immune to external influences has made it highly controversial (cf. Church, 2008). However, it should be noted that besides creating a heated polemic, FFT transformed a vague personality-and-culture problem into a directly falsifiable scientific hypothesis. Further, the theory provides guidance for future research. If some traits, or some aspects of traits, can reliably be shown to be subject to cultural influences, this raises a series of questions: Why these traits and not others? What are the relevant features of culture that produce change? By what mechanisms do they alter traits? Can these mechanisms be exploited by interventions to cultivate desirable traits?

In this article we will offer a set of hypotheses that allow empirical tests of FFT’s predictions.

### Personality determines culture

The reverse causation hypothesis—the notion that personality traits determine cultural variation—is a less-explored theoretical option, but numerous data seem to suggest that personality traits could indeed shape the cultural environment in which people live. Traits affect what music people prefer to listen to (Rentfrow and Gosling, 2003), what kinds of personal webpages they design (Vazire and Gosling, 2004; Marcus et al., 2006), how people organize their homes and offices (Gosling, 2008), and what types of cultural memorabilia they have brought back from vacation (Carney et al., 2008). Although these are rather specific habits and preferences, it seems likely that basic personality dispositions could also determine the cultural environment people create for themselves. Thus, the collective actions of groups of individuals with shared traits might modify the culture of a nation.

For example, Hofstede and McCrae (2004) assumed reversed causation in a paper in which two opposing viewpoints were presented. Based on correlations between personality and value dimensions, Hofstede and McCrae (2004) proposed that power distance—one of the Hofstede’s culture dimensions—may reflect a nation’s mean level on introversion and conscientiousness. A plausible scenario is that if there are many conscientious and introverted people in the society they will build a culture of obedience to authority, coupled with stable social institutions (Hofstede and McCrae, 2004).

### Reciprocal causality

Because cultural determination of personality does not exclude reversed causation, it is possible that causal forces operate in both directions simultaneously. This view that culture and personality can influence reciprocally each other seems to be most natural (Mendoza-Denton and Mischel, 2007). Although personality dispositions interacting with the environment shape characteristic adaptations and self-concept (McCrae and Costa, 1999), nothing seems to preclude that cultural, social, or economic environment could be molded by the elevated or lowered level of some personality trait. It is possible that systematic interactions between culture and personality can be observed. Returning to the example presented above (Hofstede and McCrae, 2004, p. 77), purposeful and determined individuals are needed to create stable and productive social institutions, but these institutions may nurture people’s conscientiousness as well.

Reciprocal causality at the level of characteristic adaptations is compatible with FFT, but reciprocal causality at the level of basic tendencies is not.

## Methodological problems

If we can replace an amorphous “personality-and-culture problem” with more specific and testable questions, that would be itself a non-trivial advancement. But before approaching these problems is necessary to consider several methodological issues.

Research on personality-and-culture is essentially observational. We normally cannot manipulate either traits or cultural institutions and must infer causal relations from the associations we observe. As with construct validation, the inference must be guided by a pattern of evidence that strengthens some interpretations and rules out others. One useful template for collecting and analyzing relevant observations is provided by Campbell and Fiske’s (1959) scheme for convergent and discriminant validation. They argued that personality traits can be validated only if two or more traits are measured each with multiple methods (or indicators); a measure of personality can be called valid only if measures of the same trait correlate more highly with each other than with measures of different traits. Variations on this scheme can be used in studies of personality-and-culture.

### Ruling out alternative explanations

As an illustration, let us suppose that extraversion was measured with the same personality scale over a 25 year period (Twenge, 2001). If the extraversion scores had increased over time, for both women and men, it would be tempting to conclude that the level of extraversion was increasing in younger birth cohorts, perhaps due to changes in the socialization process, which is one of the most powerful tools of cultural transmission. However, it is also possible that changes over time do not reflect true shifts in personality dispositions; there are plausible alternative explanations.

*Trait specificity*. One of these stems from the fact that traits are hierarchical. Extraversion is a broad disposition, typically characterized by several facets such as gregariousness, assertiveness, and positive emotions (e.g., Costa and McCrae, 1992). If only a global measure is used, it is not clear whether the core of extraversion was involved, or merely one or more of its component facets—perhaps the phenomenon is driven entirely by increases in, say, assertiveness. We need measures of several facets of the same domain to be convinced that the domain itself, not merely some component facets, was affected. The same argument occurs down the trait hierarchy: An increase in assertiveness might be driven by only one of its component nuances (McCrae, 2015)—say, self-promotion. Assertions about a trait at any level should be based on the assessment and analysis of its components, to rule out the possibility that the observed effect is specific to some of them.

*Response artifacts*. Another potential explanation, noted by Twenge (2001), is an increase in socially desirable responding, which provides an elevated temptation to describe oneself as more extraverted in order to create a more favorable self-presentation. Because the desire to create a favorable impression also dictates, for example, exaggeration of the respondent’s altruism, helpfulness, and sociability, it would be critical to see if similar changes could be observed in the scores of other evaluative personality traits. Thus, assessment of a single trait is not sufficient for distinguishing changes in the trait level from changes in the style and manner of self-presentation.

Response artifacts are a serious concern in cross-cultural comparisons. Cultures are known to differ in the tendency to acquiesce and in extreme responding as well as in socially desirable responding (Smith, 2004; Mõttus et al., 2012; Achaa-Amankwaa et al., 2021). These problems can be minimized by sound research design (e.g., the use of balanced scales to control for acquiescent responding), by statistical analyses, such as ipsatization, that correct for response styles (Rammstedt et al., 2010), and by the assessment of data quality (McCrae et al., 2005a). But in many cases, they will remain as alternative explanations of observed cultural or generational differences.

*The duality principle.* According to FFT, traits cannot be directly observed; they must be inferred from behaviors, reactions, relationships, and attitudes that are known indicators of the underlying trait. The duality principle (Costa and McCrae, 2017) asserts that the concrete thoughts, feelings, or behaviors serving as trait indicators have a dual nature: They simultaneously assess acquired characteristic adaptations and the underlying trait of which the adaptation is an indicator—just as vocabulary test scores simultaneously reflect the number of words that have been learned and the underlying ability to learn words. Because scores in personality questionnaires represent both basic tendencies and characteristic adaptations, any difference in the scores may represent both or only one of them. Consequently, if personality scores differ across cultures or historical eras, it does not necessarily mean that basic traits differ; it is possible that the observed difference lies merely in the indicators. For example, using social media is an indicator of extraversion (Bowden-Green et al., 2020), and the use of social media has increased dramatically in the past two decades, but this does not imply that extraversion has increased.

The ambiguity caused by duality is normally controlled by aggregation across a diverse set of items. During the COVID-19 pandemic, lowered endorsement of an item like “I often go to large parties” would falsely suggest a marked decline in extraversion. But a scale including items tapping both use of social media and attendance at parties would tend to cancel out these spurious effects, leaving a better assessment of extraversion. The more, and more diverse, item content in a scale, the more likely it is to accurately reflect trait levels.

Duality is a greater problem at the level of nuances. Because nuances are usually assessed by single items, it is not possible to neutralize the explicit item content by aggregation. Instead, scientific judgment may be needed: Is it more likely that the observed cultural difference is due to effects on the overt item content, or on the trait it is intended to assess? One of the items that most discriminates between cultures is “Like to stand during the national anthem” (Achaa-Amankwaa et al., 2021, p. 391). This item probably has a drastically different interpretation in totalitarian and democratic countries, and such cultural differences do not require us to assume a difference in the underlying trait. Similarly, an excitement seeking item concerning enjoyment of roller coaster riding had a very low endorsement rate in the Philippines (Church et al., 2011), not because Filipinos were truly low on this nuance, but merely because roller coasters are a relatively rare sight in the Philippines.

*Multi-trait, multi-method designs*. Besides a general strategy of identifying and assessing alternative explanations, there is also a more specific technique helping to decide whether culture has an impact on some trait or on indicators that were chosen to measure this trait. As was mentioned above, a personality trait can be defined by a common information shared by all indicators used to measure this trait (Campbell and Fiske, 1959). If we want to know how an external, also called criterion, variable is related to this personality trait then we need compare the strength of the correlations between the indicators and external variable with the strength with which the indicators represent the underlying trait [see the method of correlated vectors (Jensen, 1998)]. Thus, the logic is very simple: if we want to measure an underlying trait rather than any of its specific indicators, the strength of external correlation must be proportional to the symptomatic power—validity—of these indicators. In addition, to eliminate the measurement method problem we need some other trait, which was not subjected to the same influence, which could indicate a common method bias (cf., Podsakoff et al., 2003, 2012). These simple rules are useful tools for learning how culture can or cannot influence personality traits.

## Comments on traits and culture

### Controversies about traits

Although personality traits are real and do exist outside the minds of people who make judgments about these traits (Funder, 1995, 1999), researchers are in a permanent quest for the best possible indicators for these traits. Unlike physics, personality psychology does not have natural measurement units for personality traits, such as the speed. We may be sufficiently close to the measurement unit for the perceived pitch—*mel* (Stevens, 1975)—but we are still quite far from units for neuroticism or conscientiousness. What we can do is to identify concrete feelings, attitudes, thoughts, and habits in different environments and situations, which are proxies for these traits. Because abstract potentials or dispositions cannot be directly observed, they can only be inferred somehow from their consequences, behaviors, and the experiences that may happen during someone’s life course (e.g., McCrae and Sutin, 2018).

Although traits served as a foundations of modern personality psychology (Allport, 1937, 1966), their existence has repeatedly been questioned (Mischel, 1968; Shweder, 1991). One often-repeated argument maintains that there are no global traits; only particular or contextualized traits exist that are capable of guiding human behavior (Mischel and Shoda, 1998; Baumert et al., 2017). However, Allik and Realo (2017a) argued that it makes no sense to assume that only specific forms of home or work conscientiousness exist, whereas a generalized conscientiousness transcending many situations and contexts does not. For a physicist that might sound similar to a proposed replacement of the universal Law of Gravitation with multiple local laws of gravitations for different materials, shapes, and weights (Allik and Realo, 2017a). Aristotle erroneously believed that in free fall, bodies fall at a speed that is proportional to their weight. Because the vacuum chamber had not yet been invented, there were no tools to control for an unknown factor—air resistance—which obscured the operation of gravity. It is important to notice that no one talks about a gravitation × situation interaction when explaining why a feather falls more slowly toward the earth than a tennis ball, for instance, though many psychologists once saw interactionism as a solution for the personality-situation debate (Endler and Magnusson, 1976; Diener et al., 1984; Murtha et al., 1996).

Denial of global traits is often motivated by a pressing need to predict human behavior in everyday situations, for which global traits are not always useful. But even physicists found some everyday situations tricky to explain. For example, when shaking a container with a mixture of granules, larger particles rise to the top of the mixture (the Brazil-nut effect). Although seemingly trivial, it still requires care and imagination to learn how to apply well-known laws of physics to this particular situation (Breu et al., 2003). A lesson to be learned is that the well-known physical laws did not need a revision. What is needed is learning how to apply them to obscure situations. Similarly, if a personality theory cannot exactly predict what a person’s favorite color (Jonauskaite et al., 2021) or number is, to say nothing about their favorite combination (Simon, 1971), it does not imply that the model is wrong, or personality traits are useless. It is enough if personality psychologists can forecast, relaying on personality measures, at least some important life outcomes (Ozer and Benet-Martínez, 2006).

If there was any benefit from the person-situation debate (Kenrick and Funder, 1988), then it demonstrated how unproductive it is to think that situations alone, not people’s inclinations or dispositions, determine how people feel, think, and behave (Ross and Nisbett, 1991). Ironically, studies have demonstrated that situations themselves are best conceptualized in terms of traits. For instance, most attempts to develop a taxonomy of situations—the first step of scientific exploration—have resulted in classifications that suspiciously resemble the structure of personality traits (Guillaume et al., 2017). This is not surprising because the environment is always perceived through the lens of its affordances to human needs and aspirations (Gibson, 1979).

### Universal and near-universal in personality

Although the exact number of the basic personality traits is still debated (Ashton and Lee, 2020), no one doubts that the Five-Factor Model (FFM) of traits—neuroticism, extraversion, openness, agreeableness, and conscientiousness—offers a broad and parsimonious description of the pattern of covariations between personality traits (McCrae and John, 1992; Goldberg, 1993). Because the FFM has been shown to be applicable to most studied languages or cultures, it has been proposed that the FFM or Big Five could be human universals (McCrae and Costa, 1997).

*Universal* means that something is characteristic of all members of a class, without limit or exception (Allik et al., 2013). Because very few things exist without exceptions, the observed regularities are likely to hold not in all members of the class but in most of them. Many psychological phenomena, including personality structure, are *near universals* (Allik et al., 2013; Allik and Realo, 2017b).

Also, two forms of universality can be distinguished: there are aspects of personality that may be universal across cultures (cf., McCrae and Costa, 1997) without being universal across persons. It is widely believed that it is impossible to test whether the FFM within persons is a near universal, because the factor structure can only be derived from covariation trait-matrices in which individual data are apparently lost. It has been argued that the FFM can characterize the group as a whole, but not any individuals in that group (Borsboom et al., 2003). Yet, this is not entirely true, because a given factor structure can be extracted from covariation matrices only if a large majority of individuals have personality profiles corresponding to the same pattern of covariation (Allik et al., 2012). A good overall fit does not exclude the possibility that a small minority of participants may have an unusual configuration of personality traits, which clearly deviates from the dominant structure (Allik et al., 2018).

The pattern of covariation between personality traits or the FFM is not the only cultural near-universal. Although sex differences in the personality mean scores are small relative to individual variation within sex, these differences have a highly replicable pattern across cultures (Costa et al., 2001; Schmitt et al., 2008). Likewise, there is a cross-culturally replicable pattern of difference between how people perceive the personality of others and how they see their own personality (Allik et al., 2010).

### Dimensions of culture

The other side of the equation—culture—deserves a few words as well. The most striking property of human culture is its enormous variety. Almost every feature of culture—practices, beliefs, institutions—varies, even when comparing close neighbors. It would be a complicated or even impossible task to find out how this massive variety of cultural artifacts are related to personality. One way to cope with this overwhelming richness of details was by looking for underlying factors or dimensions. As with personality psychology (Eysenck, 1947/1998), a dimensional approach is responsible for progress in understanding human culture. Even though the idea of cultural dimensions was already discussed in mid-20th century by various social scientists, the first empirically based model of national culture dimensions was proposed by Geert Hofstede, who first distinguished four cultural dimensions—individualism/collectivism, power distance, uncertainty avoidance, and masculinity/femininity—which explained a considerable amount of the usually observed cross-cultural variance (Hofstede, 1980/2001). Hofstede later added two dimensions, long vs. short-term orientation and indulgence vs. restraint (Hofstede et al., 2010). From the four initial dimensions, individualism/collectivism enjoyed a huge popularity (Triandis et al., 1988; Triandis, 1993, 2001) because this dimension usually explained the largest proportions of variance between countries. The World Value Survey identified two dimensions—traditional/secular and survival/self-expression—as coordinates of human values (Inglehart et al., 2004). Bond with colleagues analyzed cross-cultural variation in social axioms—generalized beliefs about the functioning of oneself and the social and physical environment (Bond et al., 2004). These axioms seem to be similar to what McCrae (2009) called ethos—the manners, customs and institutions that embody the characteristic spirit of a culture. Another dimension which has attracted a considerable interest is tightness/looseness (Gelfand et al., 2011; Realo et al., 2015; Jackson et al., 2020).

Although researchers of culture have not yet reached agreement on what dimensions provide the best description of culture (Beugelsdijk and Welzel, 2018; Minkov, 2018; Kaasa and Minkov, 2022), no one seems to question that a relatively small number of abstract and directly unobservable factors are the best descriptors of cultural diversity. If the goal is to understand something substantial about the relationship between culture and personality, then it is necessary to concentrate on the underlying dimensions rather than an endless number of small details.

## Does culture determine basic traits?

As we said, there are many convincing examples of how cultural practices determine or at least influence people’s habits, beliefs, opinions, personality scripts, and schemas. However, there is less certainty in the claim that culture can determine, modify, or moderate more basic tendencies such as neuroticism or extraversion. In this part, we analyze critically evidence that can demonstrate how cultural variation can or cannot influence basic personality dispositions.

The finding that members of one culture differ from those of another on their aggregate personality profiles is clearly not proof that culture affects personality. The number of alternative explanations is large: The two groups may have different gene pools; the translations may not be fully equivalent; styles of responding, such as social desirability, may vary across cultures; samples may not be representative or comparable. Indeed, Poortinga et al. (2002) argued that the only sensible interpretation from data available today is that all differences are artifacts, and that “the distribution of scores on basic personality dispositions is the same in each culture” (p. 297). Such a view implies that culture does not affect trait levels.

But there are also many reasons to argue that observed differences are real. Church and Katigbak (2002) showed that different Filipino samples had similar profiles whether they completed English or Filipino versions of the Revised NEO Personality Inventory (NEO-PI-R; Costa and McCrae, 1992). Allik et al. (2017) found meaningful geographical ordering of self-report data from 76 cultures. McCrae et al. (2005b) showed similar aggregate personality profiles across methods of measurement (self-report vs. informant ratings) in 28 cultures. Although various artifacts doubtless distort cross-cultural comparisons to some extent, there do appear to be real substantive differences in the mean levels of traits.

It is helpful to put the effects in perspective. Allik et al. (2017) examined aggregate self-reported trait levels in 76 samples from 62 countries. Data were standardized using American norms to create *T*-scores with means of 50 and *SD*s of 10. Across the 30 NEO-PI-R facet scales, national means fell in the range of *T* = 40–60 for 98% of the cases. The average standard deviation of the mean values across all 76 samples and 30 facets was 3.46. Thus, the difference between any two cultures is about one-third of the difference between individuals within a culture. Note that this in itself goes far toward ruling out a strong form of the culture-creates-personality hypothesis: If personality were shaped entirely by culture, there would be small within-culture differences and large between-culture differences.

The question, then, is whether cultural influences create these modest personality differences. Some designs can shed light on the issue. Evidence of cultural effects could be seen in (a) the identification of qualitatively distinct traits or trait structures in different cultures; (b) personality changes with immersion in a new culture; (c) mean changes in trait level that parallel historical changes within a single culture; and (d) changes in individuals’ trait levels associated with differential culturally-salient life experiences.

### Indigenous traits and structures

Cultural and cross-cultural psychologists have often proposed that cultures have distinctive indigenous traits, such as Chinese *ren qing*, Portuguese *saudade*, Japanese *amae*, German *Schadenfreude*, or Russian *dusha* but to date, available evidence about indigenous traits is inconclusive. Allik et al. (2011) found no support for the notion that Russian personality profiles correspond to the literary and scholarly notion of a distinctive Russian soul—*dusha*. It was also proposed that Chinese culture has a unique traditional trait—interpersonal relatedness—not found in other cultures (Cheung and Leung, 1998; Cheung et al., 2001). But it was subsequently found that this trait can be found in other cultures as well, although perhaps not with the same degree of salience (Lin and Church, 2004).

A second possibility is that the same traits may appear everywhere but show a different factor structure (e.g., Durkee et al., 2022). The usual FFM structure is rather weakly replicated in many African cultures, and it might be supposed that there is a distinctly African variation on the FFM. But studies seeking a unique pan-African personality structure have not succeeded, because deviations from the universal structure seems to be erratic (Zecca et al., 2013). McCrae et al. (2005b) argued that these erratic differences may be due to error of measurement in small samples: When data from five sub-Saharan African nations were pooled, the American FFM structure was clearly replicated.

It has been observed that in one hunter-gatherer society a smaller number of personality traits was needed to describe someone’s self-reported personality (Gurven et al., 2013, 2014; Smaldino et al., 2019). Yet, it is entirely possible that the results in these studies merely reflected the lack of experience that hunter-gatherers have with personality questionnaires. For the same reason, personality structure is not as well-defined in the self-reports of children (Allik et al., 2004). The question of whether the usual structure of personality is or is not replicated in preliterate cultures remains open until informant reports are collected from external observers who are familiar with the use of Western personality questionnaires.

A third possibility is that basic traits are universal, but they have culturally unique indicators. FFT argues that culture may impact characteristic adaptations rather than dispositional traits (Church, 2010; Achaa-Amankwaa et al., 2021). McCrae (2000) proposed ethnographic studies in a wide range of cultures to identify “recurrent and culture-specific manifestations of personality” (p. 24). Intracultural researchers might use etic measures of traits to identify high and low scorers, and then conduct case studies of these individuals to see how they typically expressed their traits. These could lead to emic measures of universal traits.

### Acculturation and sojourner effects

*Acculturation*. Without any doubt, acculturation—a process in which an individual adopts, acquires, and adjusts to a new cultural environment—is the prime test case for a demonstration that culture can change personality dispositions. Therefore, it was a surprise to find only a few studies devoted to this important question. In one of the first such studies, personality traits of students of Chinese ancestry living in Canada were studied (McCrae et al., 1998). Students born in Canada were compared to recent and long-term immigrants from Hong Kong, and results suggested that exposure to Canadian culture increased openness, extraversion, and several facets of agreeableness—traits in which European-Canadians scored higher than Hong Kong Chinese. Self-report results were replicated in a second sample using peer ratings. Differences in the sense of competence and vulnerability to stress appeared to be due to different cultural standards for judging these traits (McCrae et al., 1998).

A handful of subsequent studies have provided partial support for these findings. Benet-Martínez and Karakitapoğlu-Aygün (2003) reported that second-generation Asian-Americans had scores on extraversion and openness that were intermediate between those of first-generation Asian-Americans and European-Americans. Leininger (2002) compared Vietnamese immigrants to the US who arrived when they were 9–19 years old vs. 20+ years old, hypothesizing that the younger group would be more affected by American culture. Results were mixed—for example, younger immigrants who settled in North Carolina scored higher than older immigrants in openness, but the pattern was reversed for immigrants who settled in California. Güngör et al. (2013) compared Japanese, Japanese-American, and European-American first-time mothers. To avoid problems of scalar equivalence, similarity was gauged by correlating the pattern of item responses within each of the five personality domains, and the authors concluded that the results supported the hypothesis that American culture affected the personality of Japanese-Americans. However, the results are profoundly ambiguous and say nothing about the direction of personality differences. Japanese-Americans, for example, might have had mean scale scores identical with those of Japanese, but obtained by endorsing different items. In this study, 20% of the Japanese-Americans—but none of the Japanese—were assessed in English; the similarity between Japanese-Americans and European-Americans might be due entirely to shared overlap in language.

Cultural effects on personality might be mediated by language. It has been proposed that switching languages we may also switch personalities (Ramirez-Esparza et al., 2006; Chen and Bond, 2010; Lönnqvist et al., 2014). Yet, the claim that a different language creates a different personality may be a bit of an overstatement. Bilinguals usually obtain very similar personality scores irrespective of the questionnaire’s language (Konstabel, 1999). Again, it is also necessary to distinguish changes in personality dispositions from linguistic-cultural styles for describing personality traits.

*Sojourner studies*. In recent years a number of sojourner studies have appeared (Peltokorpi and Froese, 2012; Güngör et al., 2013; Zimmermann and Neyer, 2013; Niehoff et al., 2017; Richter et al., 2021), examining the effects on personality of temporary encounters with a foreign culture. Many university students spend a semester or year abroad, and pre-post studies of trait levels provide information on the effects of that experience. Note that these are not acculturation studies, in which changes toward the normative trait profile of the host culture are hypothesized. Here, subjects typically sojourn in a variety of different cultures, with presumably different personality profiles. What all subjects have in common is only immersion for some months in a different culture. These studies address an important issue in personality-and-culture, but not the classical issue of whether personality is shaped by the specific ethos of a culture.

Clearly, a simple comparison of students who have and have not studied abroad is prone to selection biases: Pre-existing personality traits may dispose some students to study and live abroad (see Realo et al., 2023 for a recent review). All recent studies have compared sojourners with stay-at-home controls at baseline, prior to the sojourn experience, and all have reported relatively large effects: Students choosing to study abroad tend to be higher in extraversion, openness, agreeableness, and conscientiousness, and lower in neuroticism—although Söldner (2013) reported that they were lower in openness.

All these studies are pre- and post-sojourn analyses, but Söldner (2013) reported only baseline data for the control group. Without longitudinal data for controls, change due to sojourning cannot be separated from maturation—an issue in studies of young adults. Significant increases in extraversion, openness, and agreeableness and a significant decrease in neuroticism were seen in at least two of these studies, although the effects were small: Zimmermann et al. (2021) reported | *d*s| of 0.00 to 0.22 for the five factors.

These studies relied exclusively on self-reports, so the findings might be due to expectancy effects or to superficial changes in the self-concept. The latter possibility is made more plausible by a five-year longitudinal follow-up reported by Richter et al. (2021), who found that “there were no meaningful (lasting) sojourn effects on most if not all traits, except a small signal for openness” (p. 10).

Studies of acculturation or sojourner adaptation (e.g., Church, 1982) are susceptible to a wide range of confounders. If measures in two different languages are used, their comparability must be examined. If immigrants are compared to non-immigrants from the same culture, any differences might be due to self-selection effects (Söldner, 2013; Zimmermann and Neyer, 2013). To guard against such selection effects, pre- and post-immigration assessments is needed; even here, apparent changes might be due to changes in response style.

Summarizing personality acculturation studies, it would be important to emphasize the following points. First, because cross-cultural differences in personality mean scores are small compared with between-individual differences, there is a limited range for culture-caused changes. Second, the measures used must show (or be corrected to show) scalar equivalence across languages. Third, it is necessary to consider the initial or pre-departure levels of personality traits to account for selection effects. Fourth, actual change in trait levels should be separated from change in cultural standards for judging or reporting these traits (see McCrae et al., 1998). Finally, it may be necessary to separate short-term and long-term effects of acculturation, because only sustained changes reflect personality dispositions, not cultural styles, or traditions.

### Birth cohort effects

Cultures—especially modern cultures—are not static, and cultural change across decades could result in changes in collective personality. In a famous series of meta-analyses, Twenge (2000, 2001) and Twenge and Campbell (2001, 2010) have reported dramatic cohort effects on many personality traits, such as anxiety, extraversion, and self-esteem (Twenge, 2000, 2001; Twenge and Campbell, 2001, 2010). In principle, such effects might speak to the influence of changes in culture on successive generations, but it should be also noted that some of the trends reported by Twenge and colleagues have been difficult to replicate in samples that are more representative of the general population (Robins and Trzesniewski, 2005; Pullmann et al., 2009; Terracciano, 2010; Trzesniewski and Donnellan, 2010; Wetzel et al., 2018).

As was already mentioned, the observed changes in response scores may be related to response biases such as favorable self-presentation (Twenge, 2001) rather than to more fundamental changes in personality dispositions. It is also necessary to distinguish changes in personality from changes in attitudes and values, which tend to shift across generations. For example, it was noticed that social capital—the networks of reciprocal trust among people enabling society to function effectively—varies by generation although a corresponding personality trait—A1: Trust (Costa and McCrae, 1992)—appeared to change very little across time (Putnam, 2000; Schwadel and Stout, 2012).

In some designs, birth cohort effects can be studied in cross-sectional data. In one study, college-aged observers rated personality traits of Russians, most of whom were born before Nikita Khrushchev denounced repressions and cult of Joseph Stalin’s personality in 1956, and approximately 10% before Stalin’s purges in 1937 (Allik et al., 2009). These major historical events might have left their imprints on the personality of targets, and uniquely Russian cohort effects might have created a distinctive pattern of Russian age differences. Instead, age differences in general showed the same pattern seen elsewhere (Allik et al., 2009). Age differences in personality are remarkably similar in countries with quite different levels of economic development and cultural settings (McCrae et al., 1999, 2004; Costa et al., 2000; Chan et al., 2012). Deviations from the universal pattern are difficult to find, and it is even more difficult to discern what cultural or societal factors might be behind them.

### Social roles and the work environment

When individuals move from one major role status to another—from student to worker, single to married, worker to retiree—they encounter a new set of opportunities, responsibilities, and other factors that could be construed as a new subculture. In this sense, role changes are cultural changes, and their effects on personality traits, if any, would suggest a shaping effect of culture on traits.

Certainly, there are normative changes in the mean level of traits across the human lifespan—for example, young adults become less extraverted and more closed to new experiences with age (McCrae and Costa, 2003; Roberts and Mroczek, 2008). The social investment principle maintains that such normative changes occur because people develop and adjust their personality dispositions by making psychological commitments to social institutions such as marriage, family, and community (Roberts et al., 2005; Lodi-Smith and Roberts, 2007; Wrzus and Roberts, 2017). If so, one would expect personality changes to occur exclusively in individuals who in fact change roles. To assess commitment to family we can compare married and divorced individuals and the length of marriage and relationship (Lodi-Smith and Roberts, 2007). Because these variables can be measured independently of self-reported personality traits, one can ask how marriage or divorce changes personality. There is evidence, for instance, that women who have divorced become more dominant over a 16 year period than women who never divorced (Roberts et al., 2002), but overall, the literature on changes in personality associated with marriage and family is fragmented and the observed effects often puzzling in direction (Costa et al., 2019; Denissen et al., 2019).

Another promising example is the work environment (den Boer et al., 2019; Roberts and Nickel, 2021). For example, according to the trait activation theory (Tett et al., 2021), the work environment promotes personality traits that are relevant to efficient job performance. Using longitudinal data, it is possible to disentangle, partly at least, work environment effects on personality traits from personality preferences for different occupations. Holman and Hughes (2021) reported effects of job characteristics on personality, particularly effects of workload on personality change in openness, extraversion, and agreeableness.

There is also a study in which the convergent and discriminant validation principle was methodically used (Einat and Suliman, 2021). In this study, the impact of prison officers’ time on the job on two basic personality traits—conscientiousness and agreeableness—was observed (Einat and Suliman, 2021). [If a question arises about what a job in prison has to do with culture, then it is helpful to remember that the most influential model of cultural dimensions was developed on the basis of beliefs regarding work goals and values among IBM employees across 50 different countries (Hofstede, 1980/2001).] After 4 years of work, there was a clear difference between prison officers and the control group: The mean scores of the prison officers’ agreeableness and conscientiousness decreased slightly, but only on some of the facets (Einat and Suliman, 2021). From this pattern of results, it is premature to conclude that there was a systematic change in agreeableness or conscientiousness. It is more likely that changes were in some facets, not in the global factors.

At least two studies have examined cultural differences in role transitions. In many traditional cultures, adolescents are required to end education and take on adult responsibilities at an early age; in more affluent cultures there is often a period of prolonged adolescence. If personality change is driven by role requirements, individuals in late-maturing cultures should show slower personality maturation—according to *the social investment principle*, slower declines in neuroticism and slower increases in agreeableness and conscientiousness. These effects should be seen cross-sectionally. In one multi-national study, Bleidorn et al. (2013) found the predicted effects for neuroticism and conscientiousness in cultures with early job entry, but no effects for early age of marriage. In a second multi-national study, McCrae et al. (2021) examined effects of early marriage, births per thousand women aged 15–19, length of compulsory education, and per capita Gross Domestic Product on cross-sectional rate of change in the age range from 12 to 21. None of the effects predicted by the social investment principle approached significance.

### The impact of ideologies and stereotypes on personality traits

The division of Germany between East and West was a historical experiment potentially demonstrating how an ideology could change human personality. One of the explicit goals of the communist rulers of East Germany was to cultivate a new breed of humans who were free from the egoism, greed, and selfishness cultivated by the capitalist system. How successful were they in achieving this? When Angleitner and Ostendorf (2000) studied personality traits of West and East Germans after reunification they found that East Germans scored about one-fifth *SD* lower than West Germans on openness, but there were no significant differences in any of the other factors. Thus, a half-century-long attempt to nurture a new type of personality was not too successful. This is true despite obvious differences in West and East German attitudes and values that have been preserved decades after unification (Alesina and Fuchs-Schündeln, 2007; Brosig-Koch et al., 2011).

There is another interesting opportunity for the study of the impact of an ideological system on personality. Fanny Cheung and colleagues developed the Chinese Personality Assessment Inventory to measure indigenous Chinese personality (Cheung et al., 1996, 2003, 2008). It is believed that Chinese cultural tradition (e.g., Yang, 1996) forms a basis for a Chinese personality emphasizing interpersonal relatedness which appears to transcend all political and ideological differences. Nevertheless, researchers have noticed dissimilarities in personality between Singaporean and Mainland Chinese participants (Cheung et al., 2006). Also, comparative studies of people’s values have demonstrated that participants from Hong Kong and Taiwan are less likely, compared to respondents from the People’s Republic of China, to support statements like “One should give up personal interests to fulfill parental expectations” (Yeh et al., 2013). Of course, the endorsement of filial piety is only one indicator of the absence of openness—conservatism, submission to authority, and traditionality—but it seems more likely that other indicators are also pointing in the direction of conventionality in behavior and conservative outlook (Jing and Cai, 2022). For instance, it would be tantalizing to study if one of the goals of the Cultural Revolution to destroy the Four Olds—old customs, culture, habits, and ideas—has also materialized in changes of the traditional Chinese personality.

### Stereotypes

National and regional character stereotypes are shared beliefs about the personality traits of typical citizens (Realo and Allik, 2020). One might suppose that they more-or-less accurately reflect real differences in aggregate personality traits, just as gender stereotypes correspond to assessed gender differences (Löckenhoff et al., 2014). Again, one could hypothesize that they might provide expectations that would influence the self-image and perception of others, and thus self-reported and observer-rated personality assessments. In either case, there should be convergence between national character stereotypes and assessed traits—but this has not been found. Canadians, for example, define themselves as “not Americans,” and their autostereotypes are virtually the opposite of Americans’ autostereotypes (Terracciano et al., 2005). But, when participants from Canada (and the United States) were asked to rate the personality traits of a fellow Canadian (or American) whom they knew well, there was no difference in the mean profiles of personality (Terracciano et al., 2005). Again, a stereotypical Italian—friendly, warm, affectionate, easy-going—lives in Southern Italy and is believed to differ from the more cold, ambitious, and organized Northern Italian. This stereotype, shared by both Southern and Northern Italians and by foreigners, has no kernel of truth: Direct personality ratings show their actual personality profiles cannot be distinguished (McCrae et al., 2007). Similarly, neighbors of Russia think that their national characters are mirror images of their dominating neighbor, but their real personality traits are only trivially different from Russians (Realo et al., 2009).

McCrae (2009, 2017) proposed that cultures themselves could be viewed within the framework of the FFM. He used the term *ethos* to express patterns in cultural values, customs, and institutions that could be described in trait terms. For example, the strict law enforcement policies of Singapore might be seen as dutifulness; the American Protestant Ethic might be seen as culture-level achievement striving. McCrae (2017) collected ratings of American, German, Chinese, and Russian ethos from knowledgeable informants, who showed high inter-rater reliability. If culture shapes personality, we would expect that these ratings would be positively correlated with assessed aggregate personality traits (McCrae and Terracciano, 2008). But none of the four countries showed a significant correlation across the 30 facets.

However, correlations of ethos ratings with national character stereotypes were significant and substantial (0.46–0.61) for three of the cultures, and showed a trend (*r* = 0.32, *p* < 0.10) for Russia. In an earlier study (McCrae, 2009), a significant correlation between ethos and stereotypes was also found for Japan. It thus appears that cultural practices and institutions do not influence personality traits but do help shape perceptions of national character.

Although there are currently no purely objective measures of traits, there are potentially objective measures of ethos. One might, for example, take the mean number of hours in a workweek as an indicator of a nation’s achievement striving, or the amount given to charity as an indicator of altruism. Multiple indicators would be needed for each facet of ethos, and confounding variables such as economic development would need to be considered, but this strategy has potential as an approach to the study of personality-and-culture.

## The reverse causation hypothesis: Personality traits determine culture

A recent Special Issue of *American Psychologist* (Varnum and Grossmann, 2021) explored contributions of psychological variables—including personality (Götz et al., 2021)—to cultural change. History is full of examples of individuals whose traits had momentous consequences for whole nations. Rousseau’s openness led him to critique society, inspiring the French Revolution (McCrae, 1996). Nelson’s dutifulness sustained British independence during the Napoleonic Wars (Costa and McCrae, 1998). American democracy would be far different if Washington had been as ambitious as Napoleon (cf., Rubenzer et al., 2000). Granted, these individuals and their traits did not shape culture single-handedly, but it is hard to deny that they made significant contributions.

### Personality’s consequences for culture

It is surely reasonable, then, to propose that the aggregate personality of a group can create or modify its culture. We know from the expeditions of Zheng He (1371–1435 C.E.) that the Chinese could have been world conquerors if they had wished to, but it was Western Europe that eventually colonized the globe. The introversion of the Chinese and extraversion of Europeans (Allik and McCrae, 2004) may well have been contributing causes.

There is as yet little solid evidence that variation in the personality dispositions is in fact responsible for observed differences between cultures. Like direct causation, one of the main obstacles to the demonstration of reversed causation is the universality of basic personality traits. If basic traits exist in only one relatively invariant form, then to detect consequences of these small variations is a complicated exercise. No variation, no causation! Nevertheless, although the mean personality profiles across cultures are similar, the relatively small variations that still exist between them have a systematic geographic pattern (Allik and McCrae, 2004). This pattern, though, is not homologous to the distribution of values around the world (Allik et al., 2017), so it seems unlikely that population personality traits shape national values. Hofstede and McCrae’s (2004) dimensions of culture are robustly associated with aggregate personality traits, but which is cause and which effect remains unclear (Hofstede and McCrae, 2004).

Experimental methods might give some clues. Research participants might be grouped according to assessed personality—say, introverts versus extraverts—and given group tasks. Would these groups evolve replicable interaction patterns that showed the effects of collective traits on spontaneous social organization? Would extraverts form individualistic groups, and introverts collectivistic? If so, it would support the view that, over time, traits might shape culture.

### Gene flow

Because all personality traits have substantial heritability, the transfer of genetic material from one population to another—gene flow—could be a mechanism causing geographic differences in personality (Rentfrow, 2014). It has been observed that countries formed from emigrants have higher extraversion than those countries from which the emigrants originated (Lynn, 1981). Perhaps more extraverted individuals migrated, taking their genes with them.

Small populations living on islands provide a good model for the study the effects of gene flow on personality (Camperio Ciani, 2017). In a series of studies, Camperio Ciani and Capiluppi (2010), Camperio Ciani et al. (2007) and Ciani et al. (2013) demonstrated that islanders who stay have lower levels of extraversion and openness and higher levels of emotional stability and conscientiousness, traits that are all well adapted to the prevalent socio-ecological niche of a small, isolated island. Those who are more ready to escape from a monotonous life have higher levels of extraversion and openness to new experiences.

In these studies, culture and personality become more closely attuned through mechanisms of self-selection and immigration, and it might therefore be argued that culture shapes the *collective* personality of the population. But there is no evidence here that it affects traits in the individual, which is the usual premise of culture and personality studies.

### Personality dispositions required for maintaining social institutions

Interestingly, economists have entertained the idea that personality dispositions can influence cultural practices. McClelland (1961/2010) was the first to argue that a sufficiently high level of a personality trait—achievement motivation—was a necessary condition for the start of economic growth. More recently, economists have taken an interest in altruism. Because altruism seems to prevail over self-interest in all known cultures, it is tempting to conclude that it is human nature shaping social and cultural practices (Henrich et al., 2001, 2005). Strong reciprocity is a predisposition to cooperate with others and to punish those who violate the norms of cooperation. In a series of studies (Fehr et al., 2002; Fehr and Fischbacher, 2004; Fehr and Gintis, 2007; Gintis et al., 2008; Gintis and Fehr, 2012), participants played simple economic games revealing whether they considered only self-interest or also the interests of other players—the public good. In all societies studied, decisions were made to increase both self-interested material payoffs and contributions in the public-good, but there were also cross-cultural differences (Henrich et al., 2001, 2005). Based on these subtle differences, it was proposed that a sufficient number of strong reciprocators in in the population is required for building social institutions carrying the spirit of reciprocity. Modeling demonstrated that a small number of strong reciprocators who could invade a population could make strong reciprocity an evolutionarily stable strategy (Gintis et al., 2003; Fehr and Gintis, 2007). Although the culture of reciprocity seems to require individuals with an elevated level of trust and agreeableness, it can be also said that these traits are nourished by prevailing social practices and institutions: the higher the degree of market integration and the higher the payoffs to cooperation in everyday life, the greater the level of prosociality expressed in experimental games (Henrich et al., 2005). These results can be explained by culture-gene co-evolution: Culture is supposed to shape the human genome by developing dispositions of reciprocity and prosociality (Richerson et al., 2010; Chudek and Henrich, 2011).

By and large, personality psychologists have relied exclusively on questionnaire methods for studying personality-and-culture. But experimental methods, like the games used by economists, offer some advantages, because they are not susceptible to the same artifacts. Some answers may be easier to obtain by going beyond the habitual territory of personality questionnaires.

## Reciprocal determination

Because nothing seems to prevent reverse causation—people choose and create cultural environment, which matches their personality dispositions—it is likely that determination works in both directions. As mentioned above, a good model for reciprocal determination is gene-culture co-evolution (Lumsden and Wilson, 1981; Waring and Wood, 2021). Although there is no well-developed methodology for identifying reciprocal impacts, there are several phenomena that can be used as models.

### Sex differences and social development

It is tempting to believe that if women and men differ in their basic personality dispositions then it is most likely caused by the different roles they play in the society. Sex differences in personality are apparently near universal: Women report higher levels of neuroticism, extraversion, agreeableness, and conscientiousness than men across in most nations (Costa et al., 2001). Remarkably, it has been found and repeatedly replicated that the gender gap in personality traits is greater in cultures with higher human development, as measured by life expectancy, equal access to knowledge and education, and economic wealth (Schmitt et al., 2008). This surprising result contradicts social role theories, which assume that sex differences in personality traits will be attenuated or disappear in more progressive and gender-egalitarian cultures and will be pronounced in cultures with a more traditional division of roles between sexes (e.g., Eagly and Steffen, 1984; Eagly and Wood, 1999). Thus, some other sort of explanation is needed here.

One possibility is that gender differences are the same everywhere but are masked by artifacts in traditional cultures. Guimond et al. (2007) argued that in more developed nations respondents compare themselves to people in general, whereas in traditional societies men and women use their own sex as a frame of reference, thus reducing observed gender differences. When explicitly instructed to compare themselves to all other people, respondents in Malaysia (a traditional culture) showed larger gender differences.

Substantive interpretations are also possible. Schmitt et al. (2008) proposed that the widening gender gap in personality traits are caused by an interaction between intrinsic tendencies and the environment. Let us suppose that both men and women have biological and genetic predispositions, the manifestations of which are constrained or supported by the social and economic conditions in which they live. More difficult conditions in less developed human societies might suppress sex differences in personality. In more developed and egalitarian human societies, however, dispositions are not constrained in the development in their natural directions (Schmitt et al., 2008). It is relevant to notice that physiological traits, such as sex differences in blood pressure, demonstrate a similar widening gap with societal development, which is a signature of interaction (Pollard et al., 1991; Dressler, 1999).

### Self-domestication

Another example of an interaction between dispositions and the environment is a surprising decrease in violence across time. As evidence shows, the current moment is the most peaceful at least in advanced democratic countries (North et al., 2009; Pinker, 2011). Although several factors contribute to the decline of violence, Pinker (2011) believed that this could not have happened without changes in human personality. Among these changes the most remarkable are reduction of aggression, which is the primary cause of violence. Because not only emotions but also antecedents of emotional reactions seem to be relatively uniform across cultures, the largest differences are expected to be observed in the process of appraisal (Scherer, 1997; Siemer et al., 2007). One of the reasons why violence has decreased is that human beings are becoming more rational and deliberate, which seems to be achieved by better appraisal and control of emotional reactions.

Yet another factor reducing violence, pointed out by Pinker, is *feminization*. A good example of masculinity is the culture of honor in which a person feels obliged to defend their reputation by aggressive or violent behavior (Cohen et al., 1996; Cohen, 1998; Vandello et al., 2008). Typical masculine traits include the endorsement of aggression norms, overestimation of the aggressiveness of other people, and encouragement of aggression when witnessing interpersonal conflicts. A clear sign of feminization is avoiding or reducing these reactions.

Of course, the observed decrease of violence in society can happen without changes to basic personality traits. It could have been achieved by adjusting adaptation mechanisms to the current societal and cultural demands. Because the reduction of violence has been a relatively rapid process, changes in the pool of genes are presumably too slow to drive this process. Nevertheless, in one of the most outstanding experiments Dmitry Belyaev showed that the aggressiveness of wild silver foxes can be tamed in only a few generations (Trut, 1999; Dugatkin and Trut, 2017). One of the most surprising results of this experiment were changes in the physical appearance of foxes—heads became smaller, white spots appeared in the fur, tails rolled, and ears became floppy—although the only trait selected for was low aggressiveness. Because only one of a closely related group of species may became domesticated—for example, the donkey but not the zebra (e.g., Diamond, 2002)—it is possible that undomesticated species have no dispositions that can be nurtured. It is more likely that domestication presupposes a certain level of eusociality, which can be activated while aggression is “switched off” by lower levels of circulating testosterone (cf., Cieri et al., 2014).

It was noticed that these changes accompanying taming are typical to domestication in general. In addition to self-domestication—the process of adaptation of wild animals to cohabiting humans, without selective breeding of animals—humans also domesticated themselves, becoming more docile and friendly with other human beings (Hare, 2017; Wrangham, 2018; Sánchez-Villagra and Van Schaik, 2019). This means that domestication has dual consequences, on those who were targets of domestication but also those who domesticated these wild animals.

## Changing brain, changing personality

FFT acknowledges that basic personality traits can be altered by the environment, provided that it operates through modifications of their biological bases (McCrae et al., 2022). For culture, the best opportunity to change personality is by changing the brain. For example, smoking has substantial cultural differences. In Armenia over 55% of men are smokers whereas in Peru only about 7% of male population regularly inhale nicotine vapor (Reitsma et al., 2021). Some evidence suggests that besides detrimental health effects, chronic smoking affects personality traits over time, increasing neuroticism and decreasing extraversion, openness, agreeableness, and conscientiousness relative to normative changes (Lipkus et al., 1994; Stephan et al., 2019). Effects of smoking on neuroticism have been reported by other investigators (Sallis et al., 2019; Stephan et al., 2019), which may explain why a country’s smoking prevalence is related to its elevated level of neuroticism (cf., Lynn, 1971). But, neuroticism itself also predisposes for smoking (Munafo et al., 2007). Instead of a one directional path, we have a vicious circle, which sustains the whole complex.

Biologists have noticed that parasites could cause stress, which alters behavior and personality (Schaller and Murray, 2008; Barber and Dingemanse, 2010; Buck et al., 2018). It has been proposed that parasites can also modify human behavior particularly through higher responsiveness to disgust (Tybur et al., 2018). Because parasite poisoning could increase fear and decrease boldness and openness, these changes could promote ideologies of avoidance, isolation, and hate. Indeed, nations with greater parasite stress are more politically conservative, endorsing traditionalism, intergroup barriers, and negativity toward ethnic and racial outgroups (Tybur et al., 2016). A mirror side of this conservative ideology is ingroup favoritism, emphasizing familism or religiosity (Fincher and Thornhill, 2012).

Most of the effects noted here are small in magnitude, but brain damage could change personality entirely. Phineas Gage is certainly the best known example of how damage of the left frontal region could produce profound changes in personality (Damasio et al., 1994). In this regard, surgical lobotomy—in the United States alone nearly 20,000 lobotomies were performed in the mid 20th century—was the most brutal experimental intervention to change one’s personality (Gilbert et al., 2013). Considering that alcohol poisoning primarily affects the frontal lobes (Moselhy et al., 2001), one might say that experimenting with lobotomy effects on personality is still in progress. Since alcohol consumption is socially and culturally regulated, culture has a tool for changing—or preserving—personality. Malnutrition in the first years of life may also affect adult personality traits (Galler et al., 2013).

## Research hypotheses

In this article we have surveyed a wide variety of approaches to personality traits and culture and made a number of suggestions about how to design studies and interpret results. Here we offer a set of hypotheses that might stimulate future research. The central issue to be explained is why different cultures have consistently different personality trait profiles—why, for example, Americans are more extraverted than Chinese (Allik and McCrae, 2004). We propose that these differences are veridical, not artifactual, and that they can be accounted for in terms of FFT. These premises lead to a number of testable hypotheses.

H1: Cross-cultural differences in trait profiles remain after controlling for artifacts of response style.

H1 can be tested by systematically controlling the effects of acquiescence (by balanced keying), extreme responding (by ipsatization), and self-presentation (by obtaining informant ratings). Frame-of-reference effects can be controlled by providing an explicit reference group (“Compare yourself to all people in the world”) or by using items that call for a comparison of two options (“I prefer reading a book to attending a party”) instead of implicit comparisons to others (“I like parties [more than most people do]”).

H2: Cross-cultural differences in trait profiles remain after controlling for sampling biases.

H2 requires much more attention to sampling than is customary. Ideally, representative probability samples would be obtained in each culture (cf., Lima, 2002). Where that is not feasible, samples in each culture could be matched on literacy, education, age, sex, and perhaps occupation (as in Hofstede’s research on IBM employees). Where individual case matching is not feasible, such variables could be used as covariates. Meta-analyses of studies using different samples and sampling strategies (Internet, student samples, oral administration) and would lessen the chance that sampling differences account for observed profile differences.

H3: Cross-cultural differences in trait profiles remain after ruling out measurement inequivalence.

H3 concerns the quantitative and qualitative equivalence of the assessments compared across cultures. Statistical tests of scalar equivalence are commonly demanded, although they are imperfect (McCrae et al., 2005b). Administration of both versions of a scale to bilinguals, corrected for retest unreliability (McCrae et al., 1998), can provide evidence on the validity of a translation and its scalar equivalence. Boehnke (2022) argued that emic scales, in which the specific item content differs across cultures, can be shown to have scalar equivalence using an adaptation of confirmatory factor analysis. Emic scales could also be shown to be equivalent using bilinguals.

H4: Acculturation has no effect on trait levels.

FFT asserts that external influences do not affect traits. This is most straightforwardly tested by comparing recent immigrants to long-term immigrants matched on age and sex, provided several conditions are met. In essence, comparison of the two groups is like comparison of two different cultures, and the same considerations apply. Researchers must rule out the possibility that observed differences are due to response styles (which cultures may alter) or sampling biases (if self-selection pressures differed in the two periods of immigration). Measurement equivalence is also needed: Long-term Filipino immigrants to the US may be more likely to endorse enjoyment of roller coasters simply because they have tried them, whereas short-term immigrants have not. Finally, sample size must be large enough to ensure that the smallest meaningful difference (say, ω^2^ = 0.01)—if it exists—will be significant. McCrae et al. (1998) used this design and found that Chinese-Canadians born in Canada were higher in Extraversion and Openness than those born in Hong Kong, but did not differ in Neuroticism or Conscientiousness. If this finding is replicated, it argues against a strict form of FFT, and suggests a new hypothesis:

H5: Acculturation effects on trait levels are restricted to extraversion and openness.

Tests of H5 require data from a wide variety of immigrant groups, of which there is no shortage in the modern world. Ideally, data would be accumulated from many countries of origin and many countries of destination. A meta-analysis of such studies could assess whether culture affected only some traits (say, extraversion and openness), whether acculturation effects were proportional to the initial differences between groups, and whether some cultures (perhaps tighter cultures; Gelfand et al., 2011) have stronger acculturating effects than other cultures.

H6: Cultures with similar traits have similar gene pools; specifically, Nigerians, Chinese, and Indonesians will score lower than Britons and Australians on polygenic extraversion scores.

It is neither surprising nor very informative to note that the aggregate personality profiles of the United Kingdom and Australia are very similar (McCrae et al., 2005b): They share both genetic and cultural heritages. But Nigerians, Chinese, and Indonesians are all introverted relative to English and Australians; would polygenic extraversion scores show the same pattern?

H7: Cultures with similar biological environments will show similar traits; specifically, cultures with high levels of tobacco consumption will be higher in Neuroticism and lower in Conscientiousness than other cultures.

Diet, pathogen prevalence (e.g., Schaller and Murray, 2008), climate, substance usage, and a host of other features of the biological environment potentially influence personality traits; the challenge here would be to identify likely candidates worth systematic study.

## Some conclusions and prospects

Debates about the personality-and-culture relationship resemble an old parable about a group of blind men who discuss how an elephant looks after each of them has felt a different part of the elephant’s body. Analogously, those who focus on various indicators—characteristic adaptations—are convinced, rightly so, about massive cultural impacts on personality. Those who study basic dispositions are often stunned at how stable they are and how little external influences, including culture, can change them. Hence, there is no contradiction between these two seemingly opposite views. Indeed, cultural variation determines—programs, influences, or moderates—characteristic adaptations such as beliefs, habits, self-schemas, and narratives, but it has been difficult to show that it has any influence on more basic personality dispositions (McCrae and Costa, 1999, 2003, 2008, 2021). According to FFT, personality dispositions are protected from external influences because they are deeply grounded in the organism, where the forces of the psychological environmental do not easily reach.

Perhaps someday it may be possible to assess personality traits, not by questionnaires, but by objective measures, such as brain images or gene expression atlases. Until this time, the main available option is to rely on subjective self- or informant reports about specific feelings, thoughts, and behaviors. These answers are indicators based on which we can infer something about more enduring personality dispositions. Multiple indicators need to agree in pointing to the same trait from slightly different angles, to be sure that we can distinguish changes in dispositions from more superficial changes in indicators.

To bring this story to an end—there is no doubt that culture affects people’s feelings, thoughts, and behaviors. With regard to the other part—basic personality dispositions—results are fragmentary because only a few studies have followed requirements that are necessary for the measurement changes in personality traits. Available data demonstrate that even if culture can occasionally influence basic personality dispositions, the consequences are rather small. Thus, there is little evidence that culture can have a dramatic impact on the basic personality dispositions. The best chance for a change is somehow to modify the neuronal circuitry and chemistry of the brain. If these modifications involve mechanisms underlying personality dispositions, then it is likely that some personality traits will be changed. A future task is to scrutinize the relatively rare cases where insulation of personality traits was breached, granting access to a potential modification.

The field of personality-and-culture cannot be studied by randomized experiments, so the observed associations must be interpreted with great caution. Most psychologists are environmentalists and are therefore easily persuaded that national differences in aggregate personality traits must be the result of cultural and historical forces. However, there is by now sufficient reason to question that assumption. Instead, alternative explanations must be explored and ruled in or out, using a variety of methods and experimental designs. The distinction between traits and their indicators must be kept in mind; the equivalence of translations must be assessed; simulation studies (such as the group behavior of introverts vs. extraverts) may be helpful. Natural experiments, such as dividing a genetically homogeneous population into different parts, which are subjected to different sociocultural treatments, must be exploited.

Personality-and-culture studies were once conducted by anthropologists in preliterate cultures. Today we have the advantages of well developed and universal models of personality traits, interpretive principles such as construct validation and convergent and discriminant validity, and an Internet that makes multi-national collaborations easy. Given creative research designs and thoughtful analysis, we should be able to figure out the relations between personality and culture.

## Ethics statement

Ethical review and approval was not required for the study on human participants in accordance with the local legislation and institutional requirements. Written informed consent for participation was not required for this study in accordance with the national legislation and the institutional requirements.

## Author contributions

All authors listed have made a substantial, direct, and intellectual contribution to the work, and approved it for publication.
